# Transcriptome Differences in Pig Tracheal Epithelial Cells in Response to *Pasteurella Multocida* Infection

**DOI:** 10.3389/fvets.2021.682514

**Published:** 2021-08-19

**Authors:** Xueying Wang, Fei Wang, Lin Lin, Wan Liang, Songtao Liu, Lin Hua, Xiangru Wang, Huanchun Chen, Zhong Peng, Bin Wu

**Affiliations:** ^1^State Key Laboratory of Agricultural Microbiology, College of Animal Science and Veterinary Medicine, Huazhong Agricultural University, Wuhan, China; ^2^Key Laboratory of Preventive Veterinary Medicine in Hubei Province, The Cooperative Innovation Centre for Sustainable Pig Production, Wuhan, China; ^3^Key Laboratory of Prevention and Control Agents for Animal Bacteriosis (Ministry of Agriculture), Institute of Animal Husbandry and Veterinary Science, Hubei Academy of Agricultural Sciences, Wuhan, China

**Keywords:** *Pasteurella multocida*, new-born pig tracheal epithelial cells, respiratory epithelial barriers, dysfunction, RNA-Seq

## Abstract

*Pasteurella multocida* generally colonizes mammalian/bird respiratory tracts and mainly causes respiratory disorders in both humans and animals. To date, the effects of *P. multocida* infection on the respiratory epithelial barriers and molecules in host respiratory epithelial cells in their response to *P. multocida* infection are still not well-known. In this study, we used newborn pig tracheal epithelial (NPTr) cells as an *in vitro* model to investigate the effect of *P. multocida* infection on host respiratory epithelial barriers. By detecting the transepithelial electrical resistance (TEER) values of NPTr cells and the expression of several known molecules associated with cell adherens and junctions, we found that *P. multocida* infection disrupted the barrier functions of NPTr cells. By performing RNA sequencing (RNA-Seq), we determined 30 differentially expressed genes (DEGs), including the vascular endothelial growth factor A (VEGFA) encoding gene *VEGFA*, which participated in biological processes (GO:0034330, GO:0045216, and GO:0098609) closely related to epithelial adhesion and barrier functions. These 30 DEGs participated in 22 significant signaling pathways with a *p*-value < 0.05, including the transforming growth factor (TGF)-beta signaling pathway (KEGG ID: ssc04350), hypoxia-inducible factor 1 (HIF-1) signaling pathway (KEGG ID: ssc04066), epidermal growth factor receptor (EGFR) tyrosine kinase inhibitor resistance (KEGG ID: ssc01521), tumor necrosis factor (TNF) signaling pathway (KEGG ID: ssc04668), and mitogen-activated protein kinase (MAPK) signaling pathway (KEGG ID: ssc04010), which are reported to have roles in contributing to the production of inflammatory factors as well as the regulation of epithelial adhesion and barrier function in other tissues and organisms. The results presented in this study may help improve our understanding of the pathogenesis of *P. multocida*.

## Introduction

The zoonotic pathogen *Pasteurella multocida* is the causative agent of multiple diseases in a variety of domestic and wild animals and in humans (1). According to the capsular antigens, *P. multocida* strains are classified into five serogroups (A, B, D, E, and F) (1–3). In pigs, *P. multocida* strains, in particular those belonging to serogroups A and D, are commonly associated with the development of respiratory disorders, such as progressive atrophic rhinitis (PAR) and pneumonia (4). *P. multocida* also plays a crucial role in porcine respiratory disease complex (PRDC) and hemorrhagic septicemia (5). This pathogen is proposed to be a threat of high impact to the pig industry, as evidenced by its prevalence: 8.0% in diseased pigs with pneumonia or PAR in China, 10.3–15.6% in pigs with pneumonia in Korea, and 15.6% of isolated respiratory pathogens in the United States (6). However, current knowledge about the pathogenesis of swine is limited.

In mammals, respiratory tracts such as the trachea and bronchi are areas where *P. multocida* strains commonly colonize (7). The mammalian respiratory epithelium forms the first line of defense against the invasion and stimuli of respiratory pathogens (8, 9). As an important respiratory pathogen, *P. multocida* infection might induce the dysfunction of the host epithelial barriers, thereby contributing to the bacterial invasion and the development of many inflammatory disorders of the airways and lungs (9). However, the effects of *P. multocida* infection on the epithelial barriers and the responses of mammalian respiratory epithelial cells to *P. multocida* infection are still not well-known. Recently, several technologies such as RNA sequencing (RNA-Seq) have emerged as powerful tools to analyze gene expression and have been widely used in “pathogen–host interaction” studies (10). To elucidate the genes in mammalian tracheal epithelial cells in response to *P. multocida* infection, we used pig tracheal epithelial cells as a model and performed RNA-Seq to identify the differentially expressed genes (DEGs) in the cells during bacterial infection.

## Materials and Methods

### Bacterial Strains, Cells, and Growth Conditions

*P. multocida* HN05 is a serogroup D strain which was isolated from the trachea of a pig with respiratory disorders in Hunan Province, China, in 2010. We have determined its whole genome sequence and deposited it to GenBank with the accession number PPVF00000000 (1). Strain HN05 was cultured in tryptic soy broth (TSB) medium (Becton, Dickinson and Company, Sparks, MD, USA) supplemented with 5% bovine serum at 37°C for 8–12 h. Newborn pig tracheal epithelial (NPTr) cells (established following serial culture of primary cells derived from tracheal tissues and were kindly gifted by Prof. Hongbo Zhou at Huazhong Agricultural University, Wuhan, China) (11) were maintained in Dulbecco's modified Eagle's medium (DMEM; Gibco, ThermoFisher, Waltham, MA, USA) supplemented with 10% fetal bovine serum (Gibco, ThermoFisher, Waltham, MA, USA) under 5% CO_2_ atmosphere at 37°C.

### Cell Culture and Bacterial Infection

Monolayer cells (2.5 × 10^6^/2 ml per well) in each well of a six-well plate (Corning, Corning, NY, USA) were washed with phosphate-buffered saline (PBS) three times and were incubated with fresh DMEM (2 ml/well). *P. multocida* HN05 (2.5 × 10^8^/100 μl per well) was inoculated into three wells of the plate, while PBS (100 μl/well) was added into the other three wells. The plate was incubated under 5% CO_2_ atmosphere at 37°C for 4 h. The medium in each well of the plate was discarded and the cells were washed with PBS, followed by the addition of 1 ml Trizol into each well of the plate. This bacterial infection assay was performed three times at separate time points. In each independent experiment, three wells of cells were treated with either *P. multocida* or PBS. RNAs extracted from bacterial infected cells and/or PBS-treated cells in these three wells were pooled and were regarded as one sample for further use.

### RNA Isolation, Construction and Sequencing of Transcriptome Library

Total RNAs from the bacterial infected cells and the PBS-treated cells were isolated using the Trizol reagent protocol (Invitrogen, ThermoFisher, Waltham, MA, USA). The quantity and quality of the extracted RNAs were checked by using electrophoresis on 1% agarose gel and a NanoPhotometer^®^ spectrophotometer (IMPLEN, Westlake Village, CA, USA). RNA integrity was assessed using the RNA Nano 6000 Assay Kit of the Bioanalyzer 2100 System (Agilent Technologies, Santa Clara, CA, USA). After quality evaluation, a total of 1 μg RNA per sample was used as the input material for the RNA sample preparations. Sequencing libraries were generated using a NEBNext^®^ UltraTM RNA Library Prep Kit for Illumina^®^ (NEB, Ipswich, MA, USA) following the manufacturer's recommendations. Library preparations were sequenced on an Illumina Novaseq platform at Novogene Co., Ltd. (Beijing, China) using the paired-end 150-bp sequencing protocol. After sequencing, raw data in fastq format were firstly processed through in-house perl scripts. In this step, clean data were obtained by removing reads containing adapters, reads containing ploy-N, and low-quality reads from raw data. At the same time, the Q20, Q30, and GC contents of the clean data were calculated. All the downstream analyses were based on clean data with high quality. Paired-end clean reads were aligned to the reference genome (GenBank accession no. NC_010443.5) using Hisat2 v2.0.5. The mapped reads of each sample were assembled by StringTie (v1.3.3b) (12) in a reference-based approach. The data generated in this study have been deposited to NCBI's Sequence Read Archive (SRA) database, with accession number SRR14026556.

### Differential Expression Analysis

The gene expression of the cells incubated with *P. multocida* and/or PBS was analyzed using the DESeq2 R package (1.16.1). The resulting *p*-values were adjusted using the Benjamini and Hochberg approach for controlling the false discovery rate. Genes with an adjusted *p*-value < 0.05 found by DESeq2 were considered as differentially expressed.

### Quantitative Real-Time PCR

To validate the results of RNA-Seq, NPTr cells were treated as described in section Cell culture and bacterial infection, and total RNAs were extracted from both the bacterial infected cells and the PBS-treated cells. Complementary DNAs (cDNAs) were synthesized using a PrimeScript™ RT Master Mix Kit (TaKaRa, Kusatsu, Japan). The transcription of selected genes in different groups of cells was detected by quantitative real-time PCR (qPCR) using the primers listed in Table 1. The relative transcription levels of the genes are shown as a ratio of the target gene to the reference gene using the formula 2^−(ΔΔCt)^ (14).

**Table 1:** Primers for the detection of selected differentially expressed genes by qPCR.

**Primers**	**Target genes**	**Sequences (5^**′**^-3^**′**^)**	**Product size (bp)**	**Description**
Occludin-F	*Occludin*	ATCAACAAAGGCAACTCT	157	Encoding the integral tight junction membrane protein Occludin
Occludin-R		GCAGCAGCCATGTACTCT		
E-cadherin-F	*E-cadherin*	GCACCAACCCTCCTGAGTGT	69	Encoding the E-cadherin protein, which is one of the most important molecules in cell–cell adhesion in epithelial tissues
E-cadherin-R		AAAGTTTCCAATTTCATCAGGATTG		
β-catenin-F	*β-catenin*	GCCTTCACTACGGACTACC	182	Encoding the dual-function protein β-catenin involved in the regulation and coordination of cell–cell adhesion and gene transcription
β-catenin-R		ATCCTGATGAGCACGAACC		
CLDN-F	*Claudin*	GCATCATTTCCTCCCTGTT	156	Claudins are tight junction proteins involved in the establishment of barrier properties.
CLDN-R		TCTTGGCTTTGGGTGGTT		
ZNF395-F	*ZNF395*	GGGAAGGTACCTCCCCATCT	114	Zinc finger protein 395 encoding gene
ZNF395-R		ATGGTGGGAGCTGCTACCTA		
ARRDC3-F	*ARRDC3*	TGCTGTTCGAATTGCGTGT	103	Arrestin Domain Containing 3 encoding gene
ARRDC3-R		CTTCTGGAAGCTGGCTGTGA		
IER3-F	*IER3*	GAGGCTCTGGTCCCGAGATA	134	Immediate Early Response 3 encoding gene
IER3-R		GCGCCGGACCACTCG		
SLC2A1-F	*SLC2A1*	ATCATCGGTGTGTACTGCGG	150	Solute Carrier Family 2 Member 1 encoding gene
SLC2A1-R		GTCCAGGCCAAATACCTGGG		
NFKBIA-F	*NFKBIA*	CTTCTGGAAGCTGGCTGTGA	145	NFκB inhibitor alpha encoding gene
NFKBIA-R		CCTGCAGAATGGAGTGGAGG		
SGK1-F	*SGK1*	TCGTCTTCGCTCCAAAGCTT	88	Serum/Glucocorticoid Regulated Kinase 1 encoding gene
SGK1-R		ACACAGGGCTGATCACACAG		
ENC1-F	*ENC1*	CAGGACAGCGAGGTCAACTT	118	Ectodermal-Neural Cortex 1 encoding gene
ENC1-R		CCAGGAGGGATTCTGCGTTT		
FLRT2-F	*FLRT2*	CTTTGTGCTTGTGGTCCTGC	124	Fibronectin Leucine Rich Transmembrane Protein 2 encoding gene
FLRT2-R		CTTTGTGCTTGTGGTCCTGC		
LFNG-F	*LFNG*	ATGAGCAGGTGACCTTGAGC	82	LFNG O-Fucosylpeptide 3-Beta-N-Acetylglucosaminyltransferase encoding gene
LFNG-R		GCCTCCACTGAGAAAGGTCC		
PHLDA1-F	*PHLDA1*	GTAGAGCGCAAGGGCAAGTA	115	Pleckstrin Homology Like Domain Family A Member 1 encoding gene
PHLDA1-R		CCATCTGCAGCGTGATTTCG		
GAPDH-F	*GAPDH* ^a^	ACAGGGTGGTGGACCTCATG	178	Glyceraldehyde 3-phosphate dehydrogenase encoding gene
GAPDH-R		GGGTCTGGGATGGAAACTGG		

a *The primers for GAPDH were adapted from (13)*.

### Electric Cell–Substrate Impedance Sensing

The putative effect of *P. multocida* on the barrier function of NPTr cells was determined with the electric cell–substrate impedance sensing (ECIS) technology, as described previously (15). Briefly, approximately 7 × 10^4^ NPTr cells were seeded on collagen-coated, gold-plated electrodes in 96-well chamber slides (96W1E+), linked to the ECIS Zθ equipment (Applied BioPhysics, Troy, NY, USA), and continuously cultured until confluence was reached. The transepithelial electrical resistance (TEER) value was recorded to reflect the barrier function of the cells. After stable maximal TEER was reached, *P. multocida* HN05 (~10^8^ CFU) was added to the cells and the changes in the TEER values were recorded automatically by the ECIS system.

### Immunofluorescence Microscopy

Immunofluorescence microscopy analysis was performed following a previously described method (16). Briefly, the NPTr monolayers in each well (~2 × 10^5^ cells per well) of a 12-well plate (Corning, Corning, NY, USA) were treated with either *P. multocida* HN05 at 100 MOI (multiplicity of infection) or 100 μl PBS (mock). The plate was incubated at 37°C for 4 h. Next, *P. multocida*-infected cells and mock cells were washed with PBS three times and fixed with 4% paraformaldehyde (PFA) for 20 min. Afterwards, PFA was removed and the samples washed three times with PBS. The samples were then permeabilized with 0.1% Triton X-100 for 10 min, washed three times with PBS, and were further blocked with 1% bovine serum albumin (BSA) for 30 min. Next, 22 mg/ml glucine was added to the samples, which were then incubated with a primary antibody and a secondary antibody consecutively for 1 h each. After washing with PBS, the nuclei were stained and embedded in Antifade Mounting Medium with DAPI (4′,6-diamidino-2-phenylindole; Beyotime, Shanghai, China). The samples were finally stored at 4°C for further analysis.

The *P. multocida*-infected samples and control samples were incubated with β-tubulin antibody (1:200; Proteintech, Wuhan, China) as the primary antibody at 4°C overnight, followed by staining with Cy3-labeled antibody against β-tubulin (1:100; Proteintech, Wuhan, China) as the secondary antibody. The samples were analyzed using a Nikon A1 HD25 confocal laser scanning microscope. Images were analyzed using NIS-Elements Viewer 4.20 software (Nikon, Tokyo, Japan), while imaging analysis of the cell thickness was calculated using ImageJ software.

### Western Blot

NPTr monolayer cells were treated with either *P. multocida* or PBS, as described above in section Cell culture and bacterial infection. Challenged cells were lysed in RIPA buffer (Beyotime, Shanghai, China) with a protease inhibitor cocktail (Sigma-Aldrich, Burlington, MA, USA), sonicated, and then centrifuged at 10,000 × g for 10 min at 4°C. The insoluble debris was removed and the protein concentration in the supernatant measured using a BCA protein assay kit (Beyotime, Shanghai, China). The cell lysates were then separated on 10% sodium dodecyl sulfate polyacrylamide gel electrophoresis (SDS-PAGE) and transferred into polyvinylidene difluoride (PVDF) membranes (Bio-Rad, Hercules, CA, USA). The blots were blocked in 5% BSA in Tris-buffered saline with Tween 20 (TBST) for 2 h at room temperature and then incubated overnight with either β-catenin polyclonal antibody, E-cadherin polyclonal antibody, or β-actin antibodies (all from Proteintech, Wuhan, China). After washing, the blots were incubated with species-specific horseradish peroxidase-conjugated antibodies and finally visualized with enhanced chemiluminescence (ECL) reagents (Beyotime, Shanghai, China). All Western blots were densitometrically quantified using ImageJ software, and the results were analyzed as the relative immunoreactivity of each protein normalized to the respective loading control.

### Statistical Analysis

All data are displayed as the mean ± standard error of mean (SEM) and were evaluated using unpaired, two-tailed Student's *t*-test. GraphPad Prism 8.0 software was used for all statistical analyses. Significant differences were considered at *p* < 0.05.

## Results

### *P. multocida* Infection Induces the Disruption of the Barrier Functions of NPTr Cells

**Figure 1:**
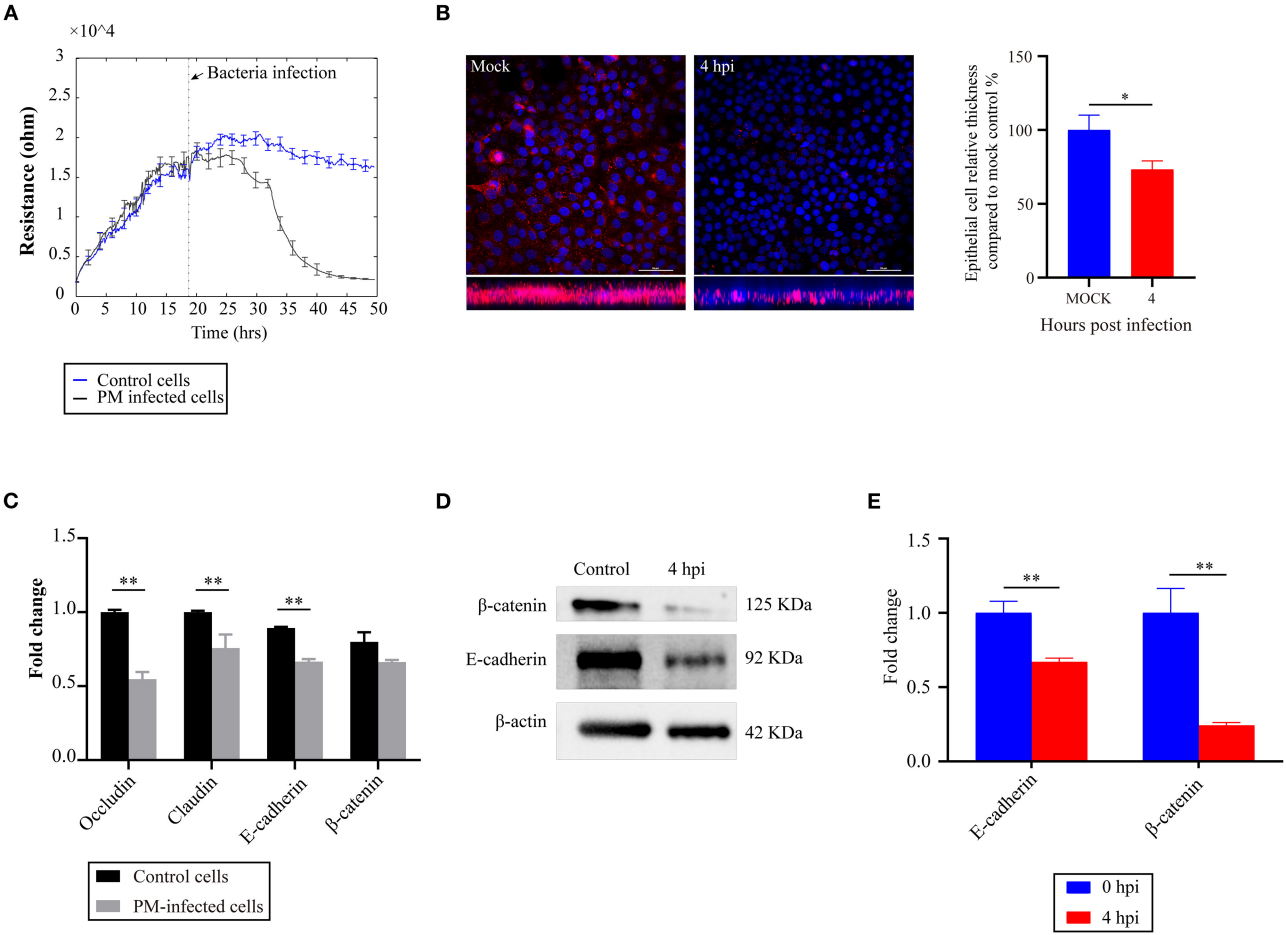
Effects of *Pasteurella multocida* infection on the barrier functions of newborn pig tracheal epithelial (NPTr) cells. **(A)** Changes in the transepithelial electrical resistance (TEER) values of the NPTr cells during *P. multocida* infection as monitored by an electric cell–substrate impedance sensing (ECIS) system. **(B)** Analysis of the density of microtubules at the cell distal area in *P. multocida*-infected cells compared to the control cells. Bacteria- or PBS-treated cells were stained with antibodies to β-tubulin (in pink under immunofluorescence microscopy). Left shows the vertical sections of the immunostained samples, while the *column chart* in the right reveals the relative thickness of the epithelial cell layer compared to mock cells. *Scale bars*, 50 μm. **(C)** Transcription of selected molecules (*Occludin, E-cadherin*, β*-catenin*, and *Claudin*) associated with cell adherens and junctions in different groups of cells as determined by qPCR. **(D)** Expressions of E-cadherin and β-catenin in different groups of cells as determined by Western blot. **(E)** Densitometrically quantified results of Western blot using ImageJ software. Data are presented as the as mean ± SEM. *p < 0.05; **p < 0.01. *PM, Pasteurella multocida*; *hpi*, hours post-infection.

To explore the effects of *P. multocida* infection on the barrier functions of the cells, we used an ECIS system to monitor changes in the TEER values. The results revealed that infection of *P. multocida* induced a significant decrease in the TEER values of the cells (Figure 1A). Analysis of the microtubule structure showed a lower density of microtubules at the cell distal area in *P. multocida*-infected cells compared to that of the control cells (Figure 1B). Since adherens and tight junctions are crucial for epithelial adhesion and barrier function in many tissues and organisms (17), we detected the transcription of several genes (*Occludin, E-cadherin*, β*-catenin*, and *Claudin*) associated with cell adherens and junctions by qPCR. The results revealed that *P. multocida* infection induced decreased expressions of these molecules (Figure 1C). The Western blot assay also showed that the expressions of E-cadherin and β-catenin were downregulated post-*P. multocida* infection (Figures 1D,E). All of the above findings indicated that *P. multocida* infection resulted in the dysfunction of the NPTr barrier functions.

### Transcriptome Sequence Data From RNA-Seq

To find the key molecules or pathways in NPTr cells mediating the dysfunction of the cell barrier functions during *P. multocida* infection, transcriptome sequencing was performed. In total, six cDNA libraries from two groups (PM_infection and Control) were sequenced, which yielded a total of 63.10 million 150-bp paired-end clean reads, varying from approximately 9.43 million to 11.15 million reads for each of the samples (Table 2). More than 97.42% had quality scores at the Q20 level, and more than 93.06% had quality scores at the Q30 level. On average, approximately 95.69% of the clean reads were mapped to the reference genome (GenBank accession no. NC_010443.5) (Table 2).

**Table 2:** Transcriptome sequence data from RNA-Seq.

**Group**	**Sample**	**Clean_reads**	**Clean bases**	**% mapped reads**	**Q20 (%)**	**Q30 (%)**	**GC percent**	**Transcript no. (FPKM > 0.1)**	**Transcript no. (0.1 < FPKM <1)**
PM_Infection^a^	Infect_1	104,327,172	7.82 G	95.56	97.59	93.43	51.50	13,391	2,461
	Infect_2	94,292,576	7.07 G	95.44	97.48	93.20	51.71	13,380	2,463
	Infect_3	105,030,792	7.88 G	95.22	97.42	93.15	52.84	13,359	2,467
Control	Control_1	108,107,896	8.11 G	95.86	97.59	93.48	51.76	13,390	2,493
	Control_2	111,467,304	8.36 G	96.26	97.68	93.59	52.19	13,459	2,567
	Control_3	107,823,840	8.09 G	95.78	97.44	93.06	51.93	13,488	2,590

a *Pasteurella multocida infection*.

**Figure 2:**
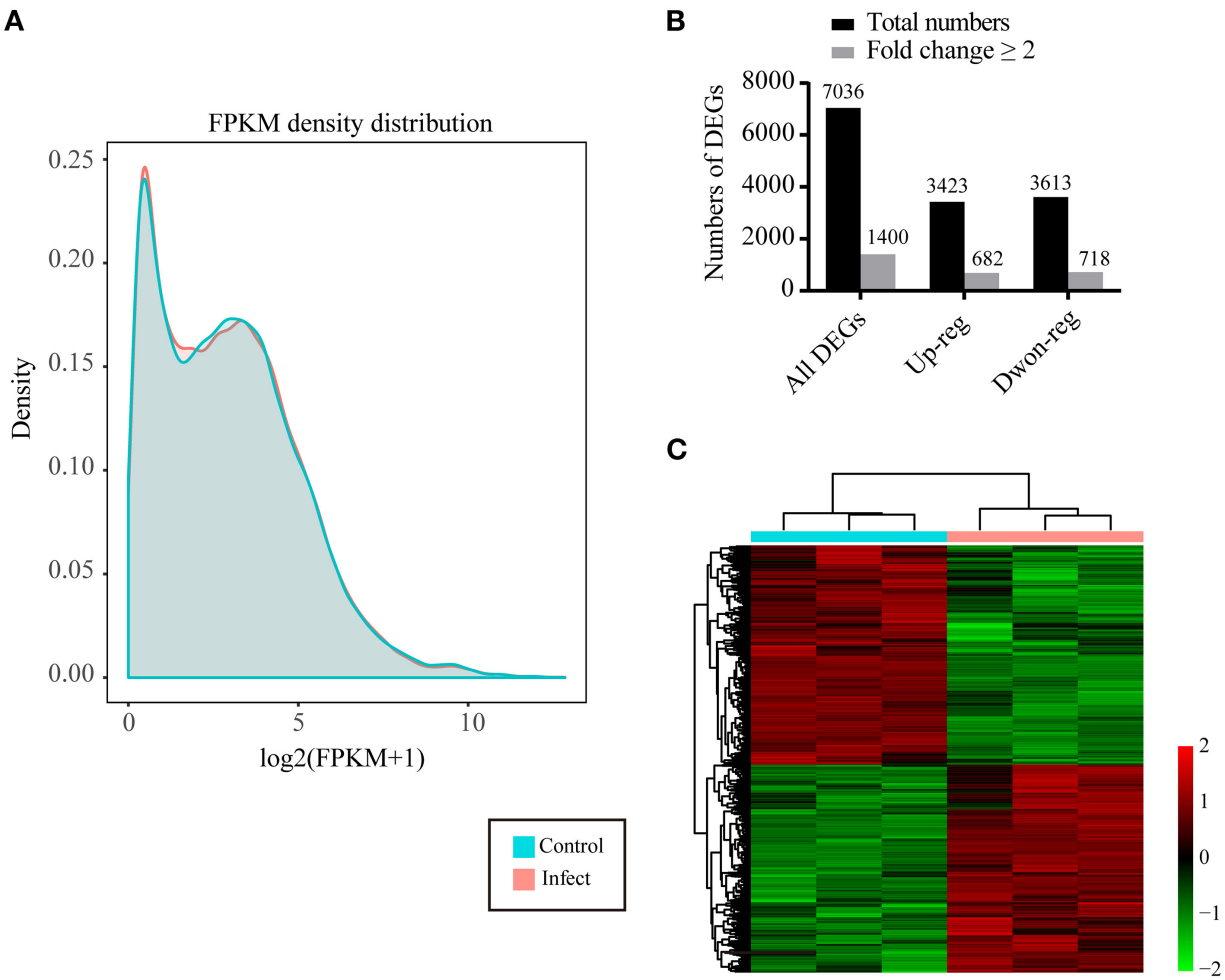
Characteristics of the differentially expressed genes (DEGs) in newborn pig tracheal epithelial (NPTr) cells infected by *Pasteurella multocida* compared to the control cells. **(A)** Density plot of a gene's log_2_(FPKM + 1) distribution visualized by CummeRbund. The *X*-axis represents the log_2_(FPKM + 1) of the genes, while the *Y*-axis represents the distribution density. Different groups are shown in *different colors*. **(B)** Number of total DEGs, upregulated DEGs, and downregulated DEGs in *P. multocida*-infected cells compared to the control cells. **(C)** Heatmap showing the distribution of the upregulated and downregulated DEGs in *P. multocida*-infected cells compared to the control cells. PM, *Pasteurella multocida*.

Calculation of the values of fragments per kilobase of transcript per million mapped reads (FPKM) identified an aggregate of 40,130 expressed genes in *P. multocida*-infected cells and 40,337 expressed genes in the control cells, with the FPKM threshold of 0.1 (Table 2). The FPKM density in the bacterial infection group and the control group exhibited a similar skewed distribution, and approximately 18.38–19.20% of the expressed genes were lowly expressed (0.1 < FPKM < 1) (Figure 2A). Compared to the control cells, a total of 7,036 DEGs were determined in *P. multocida*-infected NPTr cells [|log_2_(FoldChange)| > 1, *p*_adj_ < 0.05], including 3,613 downregulated DEGs and 3,423 upregulated ones (Figures 2B,C).

### Validation of DEGs

To validate the results of RNA-Seq, we randomly selected 10 genes, namely, five upregulated genes (*ZNF395, ARRDC3, NFKBIA, IER3*, and *SLC2A1*) and five downregulated genes (*SGK1, ENC1, FLRT2, LFNG*, and *PHLDA1*) in RNA-Seq, for qPCR detection (Table 3). The gene transcription patterns detected by qPCR were similar to those obtained from the RNA-Seq results (Table 3).

**Table 3:** Validation of the differentially expressed genes by qPCR.

**Gene name**	**Fold change**		**Description**	**Pearson's correlation coefficient**
	**qPCR**	**RNA-Seq**		
**Upregulated genes**				*R*^2^ = 0.874
*ZNF395*	12.1	11.4	Zinc finger protein 395	
*ARRDC3*	4.96	8.17	Arrestin Domain Containing 3	
*NFKBIA*	7.84	4.08	NFκB inhibitor alpha	
*IER3*	7.57	5.28	Immediate early response 3	
*SLC2A1*	1.47	4.44	Solute carrier family 2 member 1	
**Downregulated genes**				
*SGK1*	0.669	0.289	Serum/Glucocorticoid Regulated Kinase 1	
*ENC1*	0.349	0.179	Ectodermal-Neural Cortex 1	
*FLRT2*	0.559	0.240	Fibronectin Leucine Rich Transmembrane Protein 2	
*LFNG*	0.415	0.281	LFNG O-Fucosylpeptide 3-Beta-N-Acetylglucosaminyltransferase	
*PHLDA1*	0.171	0.299	Pleckstrin homology like domain family A member 1	

### DEGs Participate in the Dysfunction of Epithelial Barriers Formed by NPTr

**Figure 3:**
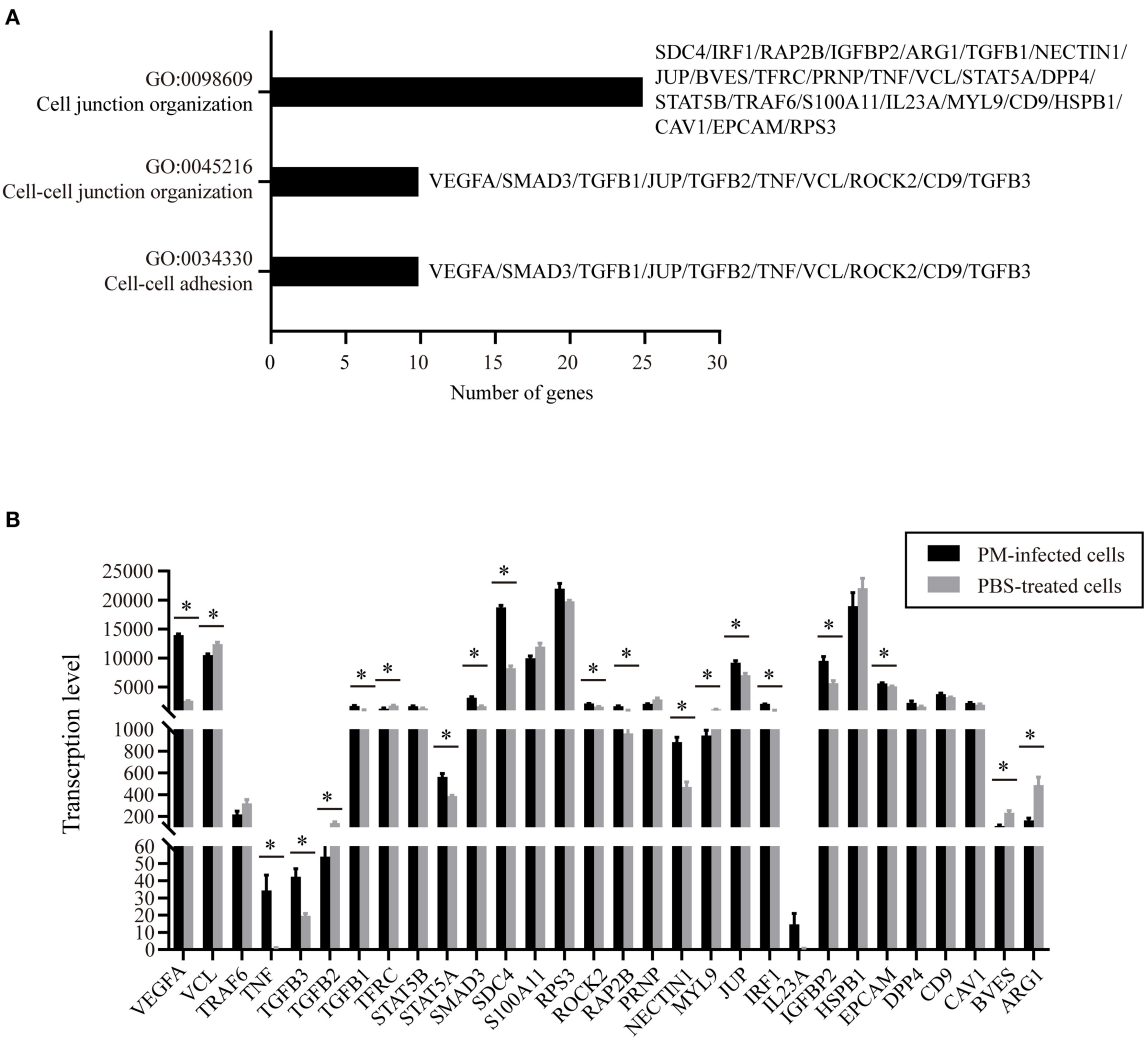
Gene ontology (GO) biological processes and differentially expressed genes (DEGs) closely related to epithelial adhesion and barrier functions. **(A)** Three biological processes (GO:0034330, GO:0045216, and GO:0098609) that are closely related to epithelial adhesion and barrier functions determined by GO analysis. **(B)** Transcription of the DEGs participating in the three biological processes in *P. multocida-*infected and PBS-treated cells as determined by RNA-Seq. PM, *Pasteurella multocida*. *p < 0.05.

**Figure 4:**
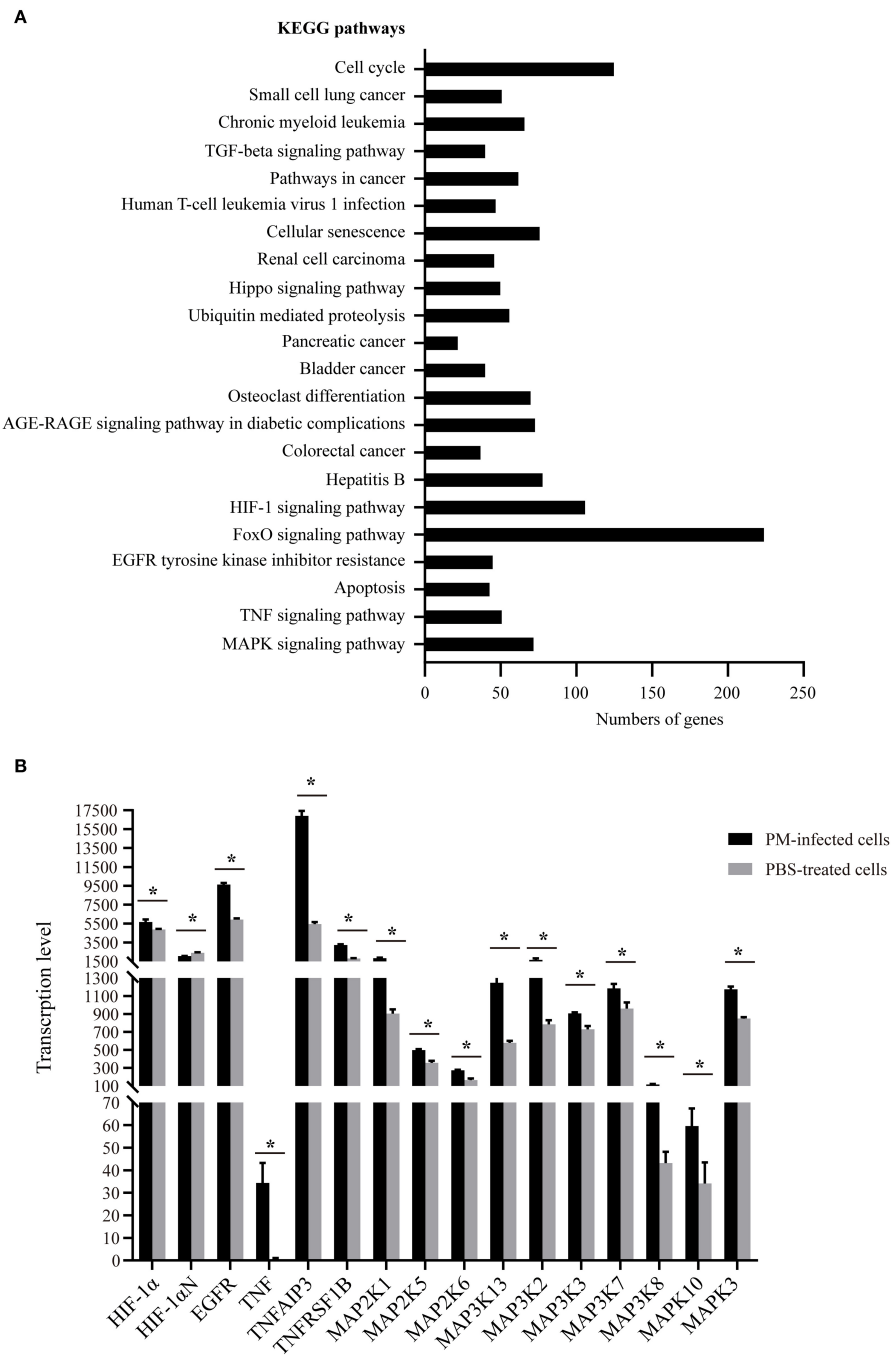
Kyoto Encyclopedia of Genes and Genomes (KEGG) pathways of the 30 differentially expressed genes (DEGs) closely related to epithelial adhesion and barrier functions. **(A)** The 22 significant signaling pathways with a *p*-value < 0.05 that the 30 DEGs participate in. **(B)** Transcription of the key genes involved in selected KEGG pathways (KEGG IDs: ssc04350, ssc04066, ssc01521, ssc04668, and ssc04010) that have roles in the regulation of epithelial adhesion and barrier functions in other tissues and organisms as determined by RNA-Seq. PM, *Pasteurella multocida*. *p < 0.05.

To understand the putative functions of the DEGs, the 6,565 DEGs determined in *P. multocida*-infected cells compared to the control cells were mapped to the Gene Ontology (GO) and Kyoto Encyclopedia of Genes and Genomes (KEGG) Orthology (KO) databases for analyses. GO analysis determined 71 enriched significant biological processes in bacterial infected cells compared to the control cells (*p*_adj_ < 0.05; Supplementary Table 1). Among these enriched biological processes, three (GO:0034330, GO:0045216, and GO:0098609) were closely related to epithelial adhesion and barrier functions, and they were involved in the participation of 30 DEGs (Figures 3A,B). According to the KEGG analysis, these 30 DEGs participated in 22 significant signaling pathways with a *p*-value < 0.05 (Figure 4A and Supplementary Table 2). Among these 22 significant KEGG signaling pathways, the transforming growth factor (TGF)-beta signaling pathway (KEGG ID: ssc04350), hypoxia-inducible factor 1 (HIF-1) signaling pathway (KEGG ID: ssc04066), epidermal growth factor receptor (EGFR) tyrosine kinase inhibitor resistance (KEGG ID: ssc01521), tumor necrosis factor (TNF) signaling pathway (KEGG ID: ssc04668), and mitogen-activated protein kinase (MAPK) have been shown in previous studies to have roles in the regulation of epithelial adhesion and barrier function in other tissues and organisms (18–21). Therefore, we checked the transcription of the key genes involved in these pathways. The results revealed that the key genes positively regulating the pathways showed significantly increased levels of transcription in bacterial infected cells compared to those in control cells (Figure 4B). In contrast, the key genes (HIF-1αN) negatively regulating the pathways displayed significantly decreased levels of transcription in bacterial infected cells compared to those in control cells (Figure 4B).

## Discussion

As a leading cause of human and animal respiratory disorders, *P. multocida* and its interaction with respiratory epithelial cells are important for pathogenesis. However, knowledge about the influence of *P. multocida* on the respiratory epithelial barrier and the interaction between the bacteria and epithelial cells is still limited. In the present study, we used NPTr cells as a model and found that infection of *P. multocida* led to decreases in the TEER values of the cells. For the culture models of the endothelial and/or epithelial monolayers, TEER is a widely accepted quantitative technique to measure the integrity of tight junction dynamics (22, 23). A decreased TEER value reflects the dysfunction of the endothelial or epithelial barriers in cell culture models (23, 24). The above finding is suggestive of *P. multocida* infection disrupting the host's respiratory barrier formed by epithelial cells. To verify this result, we investigated the thickness of the epithelial cell layer post-*P. multocida* infection through immunofluorescence microscopy analysis. In this experiment, treated cells were stained with antibodies to β-tubulin (in pink under immunofluorescence microscopy), and the results revealed that *P. multocida* infection led to a decrease of β-tubulin. It is known that β-tubulin is a major component of microtubules that perform many functions, including intracellular transport and the generation and maintenance of cellular morphology, in eukaryotic cells (25). In many studies, a change in microtubules (indicated by β-tubulin) has been used as an agent to indicate endothelial barrier functions (26, 27), and a decreased density of microtubules (indicated by β-tubulin) suggests an increased endothelial cell permeability (27, 28). Consistently, a decreased density of microtubules (indicated by β-tubulin) in *P. multocida*-infected cells may also indicate decreased endothelial barrier functions. We also detected the transcription of several known genes that encode proteins playing roles in the maintenance of the barrier functions of epithelial cells, such as *Occludin, E-cadherin*, β*-catenin*, and *Claudin* (17). The results revealed that the transcription of these genes decreased significantly during *P. multocida* infection. The Western blot assays also confirmed the decreased expressions of E-cadherin and β-catenin from the protein level (we did not determine the expressions of Occludin and Claudin because we did not have their antibodies at hand during the revision). All of the above findings suggest that *P. multocida* infection disrupts the host respiratory epithelial barrier. This process might be beneficial for the invasion of *P. multocida* and the development of pneumonic pasteurellosis, as well as the other inflammatory disorders of the airways and lungs (9).

To find out possible molecules in host cells that contribute to the dysfunction of the respiratory barrier during bacterial infection, we determined DEGs in *P. multocida*-infected cells compared to the PBS-treated cells by using RNA-Seq. Our further analyses finally determined 30 DEGs participating in three biological processes (GO:0034330, GO:0045216, and GO:0098609) that were closely related to epithelial adhesion and barrier functions. Among these genes, *VEGFA* is the most noteworthy one. *VEGFA* encodes vascular endothelial growth factor A (VEGF-A), which is known for its functions in angiogenesis, stimulating endothelial cell proliferation and migration, and increasing vascular permeability (29). An increased expression of VEGF-A has been used as an important indicator of the dysfunction of the blood–brain barrier and intestinal barrier (30, 31). Here, we found that the transcription of *VEGFA* significantly increased in *P. multocida*-infected NPTr cells, suggesting that VEGF-A might also play a role in mediating the dysfunction of the tracheal epithelial barrier after *P. multocida* infection. In other barrier models, e.g., in blood–brain barrier models, it has been shown that the production of inflammatory factors contributes to the dysfunction of the endothelial and/or epithelial barriers after pathogen infection and the activation of many signaling pathways such as TNF signaling, TGF-beta signaling, SMAD3 signaling, STAT5 signaling, HIF-1 signaling pathway, EGFR tyrosine kinase inhibitor resistance, and MAPK signaling pathway, all of which are involved in regulating the production of inflammatory factors and/or VEGF-A (32–37). The RNA-Seq results shown herein revealed that the transcription of many key factors involved in the above-mentioned signaling pathways significantly increased in *P. multocida*-infected cells, indicating that the activation of these signals might also contribute to the production of inflammatory factors, which may finally be beneficial for the dysfunction of the tracheal epithelial barrier after *P. multocida* infection.

In summary, we reported the possible effects of *P. multocida* infection on the tracheal epithelial barrier in the present study. Our findings indicate that *P. multocida* infection could induce the dysfunction of the tracheal epithelial barrier and activate several signals playing important roles in inducing the production of inflammatory factors and VEGF-A, an important marker commonly used to indicate the dysfunction of endothelial or epithelial barriers. The dysfunction of the tracheal epithelial barrier and the activation of these pro-inflammatory signals might be beneficial for *P. multocida* invasion and the development of many inflammatory disorders of the airways and lungs. Our results shown herein will provide more knowledge about the pathogenesis of *P. multocida*.

## Data Availability Statement

The datasets presented in this study can be found in online repositories. The name of the repository and accession number can be found at: National Center for Biotechnology Information (NCBI) Sequence Read Archive (SRA), https://www.ncbi.nlm.nih.gov/sra, SRR14026556.

## Author Contributions

ZP, HC, and BW contributed to conception and design of the study. XuW, FW, LL, WL, SL, and LH performed the experiments. FW and ZP performed the statistical analysis. ZP and XuW wrote the first draft of the manuscript. ZP, XiW, HC, and BW revised the manuscript. All authors contributed to manuscript revision, read, and approved the submitted version.

## Conflict of Interest

The authors declare that the research was conducted in the absence of any commercial or financial relationships that could be construed as a potential conflict of interest.

## Publisher's Note

All claims expressed in this article are solely those of the authors and do not necessarily represent those of their affiliated organizations, or those of the publisher, the editors and the reviewers. Any product that may be evaluated in this article, or claim that may be made by its manufacturer, is not guaranteed or endorsed by the publisher.

## Supplementary Material

Supplementary Table 1 Differentially expressed genes involved in 71 enriched significant biological processes in *P. multocida*-infected NPTr cells compared to PBS-treated control cells (*p*_adj_ <0.05).

Supplementary Table 2 Significant KEGG signaling pathways with a *p*-value < 0.05 that differentially expressed genes closely related to the epithelial adhesion and barrier function participate in.
